# Parental migration and cyberbullying victimization among Chinese left-behind children: understanding the association and mediating factors

**DOI:** 10.3389/fpubh.2024.1194940

**Published:** 2024-02-22

**Authors:** Menmen Wang, Jiaxue Lou, Xiaoliang Xie, Guanlan Zhao, Hui Zhu

**Affiliations:** ^1^Institute of Social Medicine, School of Medicine, Zhejiang University, Hangzhou, China; ^2^School of Public Health, Lanzhou University, Lanzhou, China

**Keywords:** parental migration, cyberbullying victimization, left-behind children, parent-child communication, internet use

## Abstract

**Introduction:**

Parental absence is greatly associated with school bullying victimization of left-behind children (LBC) in migrant families. With the increasing popularity of the Internet, little is known about the association between parental migration and cyberbullying victimization, and potential mediators.

**Methods:**

We conducted a cross-sectional study in Anhui and Zhejiang Province, China, in 2020. With a sample of 792 currently left-behind children (CLBC), 541 previously left-behind children (PLBC), and 628 never left-behind children (NLBC), path analysis was used to explore the association between parental migration and cyberbullying victimization among children, while considering the independent and sequential mediating roles of parent-child communication, and time spent online.

**Results:**

The prevalence of cyberbullying victimization was 29.3% among CLBC, 29.2% among PLBC, and 23.4% among NLBC. Path analysis showed that current left-behind status was positively associated with cyberbullying victimization among children (*p* = 0.024). Furthermore, current left-behind status was associated with worse parent-child communication, which, in turn, predicted a higher prevalence of cyberbullying victimization [95% CI = (0.007, 0.036)]. Similarly, the previous left-behind experience was associated with worse parent-child communication, which, in turn, predicted a higher prevalence of cyberbullying victimization [95% CI = (0.013, 0.043)]. Current left-behind status was associated with increased time spent online, which, in turn, predicted a higher prevalence of cyberbullying victimization [95% CI = (0.013, 0.038)]. Additionally, the current left-behind status positively predicted cyberbullying victimization among children through the serial mediating roles of parent-child communication and time spent online [95% CI = (0.001, 0.006)]. Similarly, previous left-behind experience positively predicted cyberbullying victimization among children through the serial mediating roles of parent-child communication and time spent online [95% CI = (0.002, 0.007)].

**Discussion:**

We propose that to protect CLBC and PLBC from cyberbullying victimization, it is of great importance for migrant parents to regulate children's time spent online and promote daily parent-child communication.

## 1 Introduction

China's urbanization and industrialization process continued to accelerate since 1978, and a vast number of people moved from rural areas to urban areas to earn a better living. However, most of these migrants chose to leave their children at home because of the household registration system (*hukou*) in China. Accordingly, their children are called left-behind children (LBC), defined as children whose fathers or mothers or both parents migrate to another city outside of their original residence area as recorded on the *hukou* system for at least 6 months (1). According to the data of the National Bureau of Statistics of China and UNICEF, there were over 66 million LBC in China by 2020 (2). As addressed by one recent meta-analysis, LBC were negatively affected by the migration of their parents and had greater risks of health outcomes, including anxiety, depression, conduct disorder, and suicidal ideation (3). Recent studies revealed that the adverse effects of parental migration on LBC still exist even after parent-child reunion (4). Therefore, the present study not only focused on children who were currently left-behind but also on those who were previously left-behind.

Along with the burgeoning development of computers and information technology, the Internet has been increasingly popular among children and adolescents worldwide. According to a report by the China Internet Network Information Center (CNNIC) (5), the number of underage netizens in China has reached 183 million, and the Internet penetration rate of underage netizens is 94.9%, which is even higher than that of adults. Furthermore, above four-fifths of underage netizens possess their own Internet access devices. While the increasing popularity of the Internet might be promising for child development (6), it can also present risks, and cyberbullying is one of them (7). The term “cyberbullying” was first coined in 1999 and referred to “any behavior performed through electronic or digital media by individuals or groups that repeatedly communicates hostile or aggressive messages intended to inflict harm or discomfort on others” (8). Unlike traditional ones like school bullying, cyberbullying often features anonymous and is more prevalent (9). A systematic review based on 63 studies showed that the prevalence of cyberbullying victimization ranged from 14.0 to 51.9% in children and adolescents worldwide (10).

Cyberbullying victimization is often combined with physical and psychological health damage. Cyberbullying victimization is reported to be an important predictor of body image dissatisfaction, pathological dieting behaviors, and low life satisfaction (11). Cyberbullying victimization is also associated with higher psycho-social difficulties (12), self-harm, and suicidal behaviors (including suicidal ideation, suicide plans, and suicide attempts) (13, 14). With children spending more time online due to COVID-19 in recent years (15, 16), it is urgent to pay more attention to cyberbullying victimization among LBC.

## 2 Literature review

### 2.1 Parental migration and child cyberbullying victimization

Parental absence is greatly associated with school bullying victimization of LBC in migrant families. As noted, LBC were at significantly higher risks of school bullying victimization compared with their counterparts who have never been left-behind (17). Zhang et al. found that above 30% of LBC reported being recurrently bullied (18). Additionally, being left-behind by migrant parents may further exaggerate the effects of bullying victimization on life satisfaction among children and adolescents (19). However, newly emerging forms of bullying victimization other than school bullying victimization, for instance, cyberbullying victimization among LBC, is yet to explore.

### 2.2 The mediating role of parent-child communication

Bronfenbrenner's ecological systems theory (20) underscores the family as the most influential microsystem in child development. Extensive research yields compelling evidence for the critical role of parents in children's experiences of cyberbullying victimization. On one hand, parents influence children's susceptibility to cyberbullying through their parenting practice and oversight. For instance, a study involving Romanian adolescents demonstrated that a supportive parenting style serves as a protective factor, shielding adolescents from cyberbullying victimization (21). Similarly, Wu et al. found that parental rejection from both fathers and mothers increases children's risk of experiencing cyberbullying, slowing down the decline in such victimization over time (22). Parental neglect also amplifies the risk of children being exposed to cyberbullying (23). On the other hand, the quality of parent-child relationship is a key predictor of children's vulnerability to cyberbullying. Adolescents who have a more positive and satisfying relationship with their parents are less likely to experience cyberbullying (21, 24). Liu and Chen found that parent-child closeness plays a crucial role in reducing the risk of adolescents being victims in cyberbullying (25). As primary actors in family function, effective parent-child communication also serves as an essential protector of LBC's cyberbullying victimization (10, 26). Parent-child communication enhances adolescents' social skills, such as adequate coping and social support networks (27). However, due to parental migration, the form and quality of parent-child communication changed because of the broken family structure for LBC (28, 29). Despite these findings, studies examining the effects of parent-child communication on the association between parental migration and children's cyberbullying victimization remain scarce in China.

### 2.3 The mediating role of time spent online

Self-determination theory (30–32) has been widely used to explain individual's intrinsic motivation for internet usage. According to this theory, individuals possess three fundamental psychological needs: autonomy, competence, and relatedness. The need for relatedness specifically refers to the longing for connection with others—to love and care for others, as well as to be loved and cared for in return. For LBC who are unable to live with their parents, a lack of intimate parent-child interaction might drive children to continually check messages on their Internet-connected devices (33) and to interact with others through social networking sites (34), potentially increasing their vulnerability to cyberbullying due to extended time spent online. Routine activity theory offers valuable insights for understanding diverse patterns of individual victimization (35). When children have more opportunities to access the internet, i.e., availability, their likelihood of experiencing cyberbullying also increases. Previous literature has highlighted that children who spent more time online were more likely to experience cyberbullying (36). One study conducted in Vietnam revealed the dose-response association between the average daily time spent on the Internet and the risk of experiencing cyberbullying (37). Furthermore, capable guardians play a pivotal role in influencing children's vulnerability to cyberbullying. Legate et al. found that cyberbullying can be reduced if parents discipline children by restricting their Internet access (38). Likewise, Sasson and Mesch reported that the restrictive measures taken by parents would prominently reduce the risks of cyberbullying victimization among children (39). In China, LBC tend to live with their grandparents or other relatives, especially those with both parents migrating (40), which leads to weakened parental supervision (41, 42). It is reasonable to infer that LBC may be more vulnerable to becoming victims of cyberbullying due to their increased time spent online.

### 2.4 The serial mediating roles of parent-child communication and time spent online

Positive parent-child communication plays a crucial role in fulfilling children's psychological needs, consequently reducing their dependence on electronic devices. Conversely, inadequate parent-child communication can lead to an increased time spent online among children. According to the compensatory satisfaction theory of pathological internet use (43), if children experienced low levels of psychological need satisfaction in real life, they may turn to excessive internet use. Multi-family group therapy aimed at addressing internet addiction in adolescent has shown substantial reductions in internet usage time by enhancing parent-child communication (44). Moreover, the style of parental mediation significantly influences adolescents' exposure to cyberbullying victimization on social media (45). Parents can actively manage their adolescents' online activities using strategies such as co-use or restriction (46). Additionally, parental monitoring of children's activities can bolster the cultivation of their self-control and prosocial behavior, thereby reducing the likelihood of involvement in cyberbullying victimization (47).

### 2.5 The current study

Based on the aforementioned literature, we may infer that a combination of worsened parent-child communication and lengthened children's time spent online may put LBC at a greater risk of cyberbullying victimization; however, this topic is seldom explored in China. Therefore, the current study focused on currently left-behind children (CLBC) and previously left-behind children (PLBC) and advanced the knowledge. Four hypotheses were proposed.

H1: Current left-behind status or previous left-behind experience positively associated with cyberbullying victimization among children.

H2: Parent-child communication mediates the relationship between current left-behind status or previous left-behind experience and cyberbullying victimization among children.

H3: Time spent online mediates the relationship between current left-behind status or previous left-behind experience and cyberbullying victimization among children.

H4: Parent-child communication and time spent online serially mediate the relationship between current left-behind status or previous left-behind experience and cyberbullying victimization among children.

**Figure 1:**
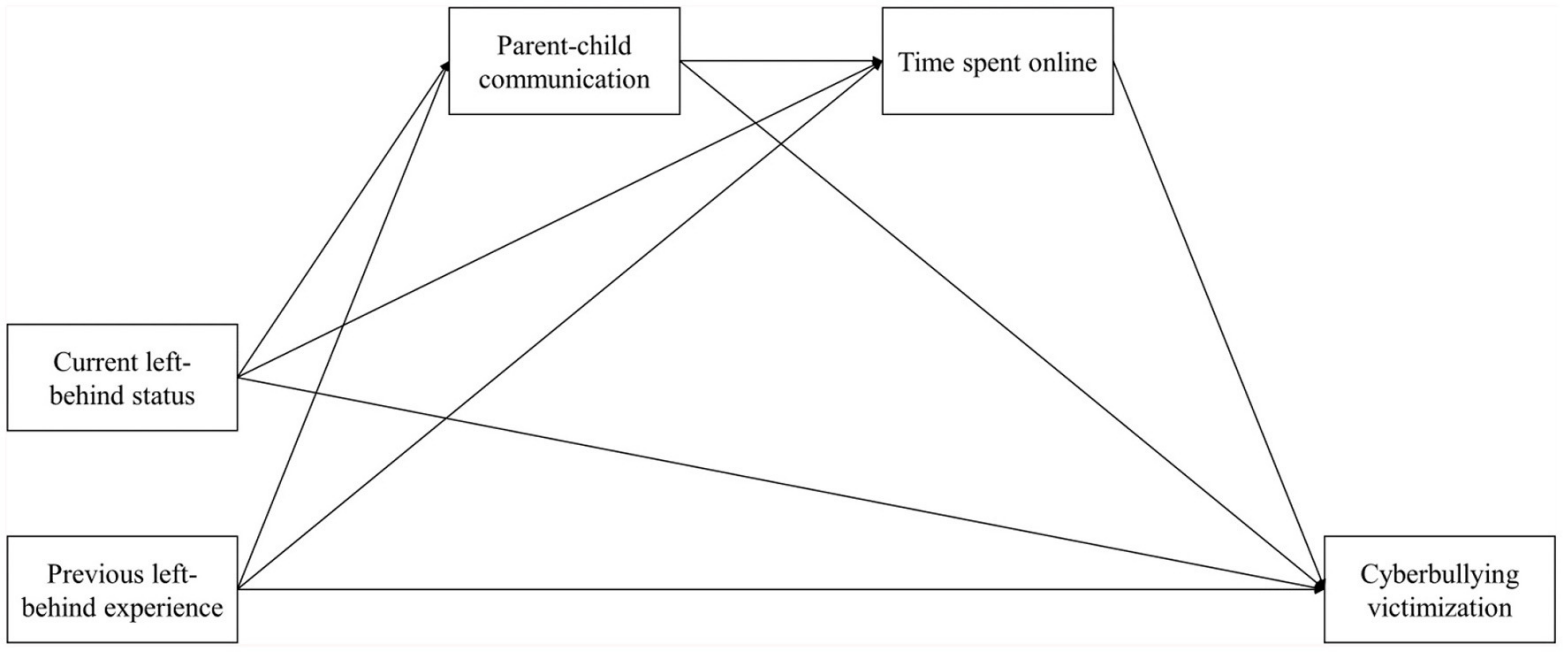
Hypothesized model of parental migration, parent-child communication, time spent online, and cyberbullying victimization.

Figure 1 shows the hypothesized research model of the present study.

## 3 Methods

### 3.1 Data collection

This was a cross-sectional questionnaire-based survey conducted in Anhui and Zhejiang province, China, from November to December 2020. Anhui Province is one of the provinces in which the number of LBC exceeds two million (2). Although Zhejiang is a national economy developed province, there is a large proportion of LBC in Kaihua County. A school-based multistage sampling method was employed to select the study population. First, three townships from a total of eight rural townships within Nanling County and three townships from a total of nine rural townships within Kaihua County were selected. Then, a primary school, and a junior high school with the highest proportions of LBC students were selected from a complete list of all schools in each township. To be eligible for this study, students need to be in Grade 5–8 (mostly including children aged 10–14) from the selected schools, and 1–4 classes were randomly selected from each school. Details of this survey were described in our previous paper (48). There were twelve schools enrolled in the present study (six primary schools and six middle schools). Of 3,025 eligible students, 2,931 completed the questionnaire, representing a response rate of 96.7%. Among these, 486 students were excluded for not meeting inclusion criteria (i.e., either parent of the child was divorced, remarried, or deceased); 90 questionnaires were identified as invalid for lack of key information about parental migration trajectories; 394 students were excluded for never accessing the Internet. Therefore, 1,961 questionnaires were included for the final analysis.

### 3.2 Measures

#### 3.2.1 Dependent variable

The Cyber Victim and Bullying Scale (CVBS) (49), a 9-item validated tool (50), was employed to assess children's cyberbullying victimization. We limited the period of cyberbullying experiences to this semester (since September 2020) to minimize recall bias. Responses included “never,” “1–2 times,” “3–5 times,” and “more than 5 times.” Students who reported they had experienced any form of cyberbullying victimization at least once during this semester were classified as the victims of cyberbullying. The Cronbach's α coefficient for the CVBS in this study was 0.84. The results of confirmatory factor analysis indicated a good fit of the CVBS to the data (RMSEA = 0.057; CFI = 0.975; SRMR = 0.024), with standardized factor loadings ranging from 0.481 to 0.684.

#### 3.2.2 Independent variables

Parental migration status was assessed with the following questions: “Did your father ever migrate to another place ever since you were born?” and “Did your mother ever migrate to another place ever since you were born?” Responses included “Yes, currently migrating,” “Yes, previous migrated,” and “No, never migrated.” Students who reported “Yes, currently migrating.” to either question were determined as CLBC. Those who responded “No, never migrated.” to both questions were identified as NLBC. The remaining were defined as PLBC.

#### 3.2.3 Mediators

Parent-child communication was accessed by the Parent-Child Communication Scale (PCCS) (51). It is a 20-item tool with good reliability and validity among Chinese children and adolescents (4, 52). The scale comprises two dimensions: communication openness and communication problems, each containing 10 items. One represents “strongly disagree” while five represents “strongly agree.” After reverse scoring the 10 items related to communication problems, the scores are summed with those of the 10 items related to communication openness. Father-child communication and mother-child communication were independently assessed, and the total parent-child communication score was derived by summing the scores of father-child communication scale (FCCS) and mother-child communication scale (MCCS). The higher the total score, the better parent-child communication. In this study, the Cronbach's α coefficients for the parent-child, father-child, and mother-child communication scales were 0.94, 0.92, and 0.90, respectively. The results of confirmatory factor analysis demonstrated a good fit for both the FCCS (RMSEA = 0.059; CFI = 0.937; SRMR = 0.072) and the MCCS (RMSEA = 0.059; CFI = 0.923; SRMR = 0.079). The standardized factor loadings for the FCCS varied between 0.509 and 0.814, and for the FCCS ranged between 0.446 and 0.782.

The children's time spent online was measured using two questions: “How many times on average have you spent online on weekdays/weekends?” with the six-point scale (1 = < 0.5 h, 2 = 0.5–1 h, 3 = 1–2 h, 4 = 2–3 h, 5 = 3–5 h, 6 = more than 5 h). We calculated the total time spent online in a week by multiplying the average hours of weekdays by five and adding the average hours of the weekend by two based on students' answers to these two questions. We took the median for each option, and for the last option, we counted it as “5 h.”

#### 3.2.4 Social-demographic variables

Socio-demographic characteristics included province, gender, age, grade, only-child status, family economic level, and parent's education level. Family economic status was measured by asking “how do you feel your family economic status compared with others in your community?” (53).

### 3.3 Data analysis

Chi-squared (χ^2^) tests and one-way analysis of variance (ANOVA) were employed to compare sample characteristics among CLBC, PLBC, and NLBC including social-demographic characteristics, parental migration status, cyberbullying victimization, parent-child communication, and time spent online. All analyses were performed using SPSS 25.0 and assumed a statistical significance level of *p* < 0.05.

We performed confirmatory factor analyses to evaluate the structural validity of the scale for the variables. To explore the mediating effects of parent-child communication and time spent online, we conducted path analysis using the maximum likelihood method. We performed 2,000 bootstraps and determined the presence of a mediating effect based on whether the confidence intervals included zero (54). The hypothesized integrated model was tested using Mplus 8.3.

## 4 Results

### 4.1 Sociodemographic

Among 1,961 participants, 792 (40.4%) were CLBC, 541 (27.6%) were PLBC, and 628 (32.0%) were NLBC. Descriptive statistics for the measurements are presented in Table 1. Males (53.1%) slightly outnumbered females (46.9%), and there were more students in middle school (57.1%) than in primary school (42.9%). More than two-thirds of students had siblings (71.4%). The majority of students came from families with fair (66.9%) or wealthy (25.5%) economic status. More than 80% of students reported their fathers' education level to be middle school or above, and more than 70% of students reported their mothers' education to be middle school or above.

**Table 1:** Descriptive statistics of sample characteristics by parental migration, *n* (%).

**Variables**	**Total**	**CLBC**	**PLBC**	**NLBC**
	***N*** = **1,961**	***n*** = **792**	***n*** = **541**	***n*** = **628**
**Province**				
Anhui	889 (45.3)	361 (45.6)	350 (48.4)	384 (42.4)
Zhejiang	1,072 (54.7)	466 (54.4)	308 (51.6)	401 (57.6)
**Gender**				
Male	1,017 (53.1)	426 (55.0)	278 (52.8)	313 (50.9)
Female	899 (46.9)	348 (45.0)	249 (47.2)	302 (49.1)
**Age, Mean (SD)**	12.04 (1.17)	12.03 (1.19)	12.14 (1.18)	11.98 (1.13)
**Grade**				
5th−6th	841 (42.9)	350 (44.2)	219 (40.5)	272 (43.3)
7th−8th	1,120 (57.1)	442 (55.8)	322 (59.5)	356 (56.7)
**Only child**				
Yes	559 (28.6)	248 (31.4)	156 (29.0)	155 (24.8)
No	1,394 (71.4)	543 (68.6)	382 (71.0)	469 (75.2)
**Family economic status**				
Wealthy	499 (25.5)	183 (23.2)	129 (23.9)	187 (29.8)
Fair	1,308 (66.9)	535 (67.8)	370 (68.5)	403 (64.3)
Poor	149 (7.6)	71 (9.0)	41 (7.6)	37 (5.9)
**Paternal education level**				
Primary school or below	356 (18.2)	142 (18.1)	105 (19.5)	109 (17.4)
Middle school	1,133 (58.1)	475 (60.4)	318 (59.1)	340 (54.2)
High school or above	462 (23.7)	169 (21.5)	115 (21.4)	178 (28.4)
**Maternal education level**				
Primary school or below	562 (28.8)	244 (31.0)	165 (30.6)	153 (24.4)
Middle school	1,032 (52.8)	419 (53.2)	283 (52.4)	330 (52.6)
High school or above	360 (18.4)	124 (15.8)	92 (17.0)	144 (23.0)

CLBC, children who are currently left-behind; PLBC, children who were previously left-behind; NLBC, children who are never left-behind.

### 4.2 Research variables

The prevalence of cyberbullying victimization in nine forms is shown in Table 2. Overall, 27.4% of students reported cyberbullying victimization during this semester. Being posted mean or hurtful comments online was the most common form of cyberbullying victimization (13.7%), followed by being excluded from online communication or games on purpose (9.6%).

**Table 2:** Cyberbullying victimization of participants (*N* = 1,961).

**Items**	***n* (%)**
(1) Someone spread rumors about me online.	110 (5.6)
(2) Someone posted mean or hurtful comments about me online.	268 (13.7)
(3) Someone threatened to hurt me online.	83 (4.2)
(4) Someone posted or used my private information online without permission.	133 (6.8)
(5) Someone posted or used my picture or video online without permission.	135 (6.9)
(6) Someone excluded me from online communication or games on purpose.	187 (9.6)
(7) Without my permission, someone got my password and sent messages to others in my name, make me lose face or cause trouble with my acquaintances.	107 (5.5)
(8) Someone sent an infected file/program to me online, or used Internet for fraudulent act.	133 (6.8)
(9) Someone used sexual symbols while chatting on the Internet.	177 (9.0)
**Victims** ^**a**^	**537 (27.4)**

^a^Children who reported they had experienced any forms of cyberbullying victimization at least once during this semester was classified as the victims of cyberbullying.

Table 3 shows mother-child communication, father-child communication, time spent online on weekdays and weekends, and the prevalence of cyberbullying victimization among CLBC, PLBC, and NLBC, respectively. CLBC reported lower scores on mother-child communication (58.25 ± 15.20 vs. 60.49 ± 14.90) and father-child communication (61.08 ± 15.32 vs. 62.56 ± 15.83) than NLBC. And PLBC reported lower scores on mother-child communication (57.49 ± 15.55 vs. 60.49 ± 14.90) and father-child communication (58.94 ± 16.27 vs. 62.56 ± 15.83) than NLBC. Compared with NLBC, CLBC and PLBC spent more time online on both weekdays and weekends. The prevalence of cyberbullying victimization was higher among CLBC (29.3%) and PLBC (29.2%) than NLBC (23.4%).

**Table 3:** Descriptive statistics and intergroup difference comparison for the variables of interest by parental migration status, *n* (%).

**Variables**	**CLBC**	**PLBC**	**NLBC**	**F/χ^2^**	***p*-value**
	***n*** = **792**	***n*** = **541**	***n*** = **628**		
**Parent-child communication** ^a^ **, Mean (SD)**					
Mother-child communication	58.25 (15.20)	57.49 (15.55)	60.49 (14.90)	6.389	0.002
Father-child communication	61.08 (15.32)	58.94 (16.27)	62.56 (15.83)	7.686	< 0.001
**Time spent online** ^b^					
**Weekday**				26.532	0.003
< 0.5 h	369 (46.6)	251 (46.4)	327 (52.1)		
0.5–1 h	167 (21.1)	138 (25.5)	149 (23.7)		
1–2 h	119 (15.0)	79 (14.6)	97 (15.4)		
2–3 h	55 (6.9)	33 (6.1)	19 (3.0)		
3–5 h	37 (4.7)	21 (3.9)	18 (2.9)		
>5 h	45 (5.7)	19 (3.5)	18 (2.9)		
**Weekend**				31.271	0.001
< 0.5 h	118 (14.9)	87 (16.1)	105 (16.7)		
0.5–1 h	173 (21.8)	129 (23.8)	173 (27.5)		
1–2 h	173 (21.8)	121 (22.4)	155 (24.7)		
2–3 h	98 (12.4)	79 (14.6)	90 (14.3)		
3–5 h	109 (13.7)	62 (11.3)	47 (7.5)		
>5 h	122 (15.4)	65 (12.0)	58 (9.2)		
**Cyberbullying** ^c^					
Victims	232 (29.3)	158 (29.2)	147 (23.4)	7.347	0.025

^a^Parent-child communication were accessed by Parent-Child Communication Scale (PACS).

^b^An interval value only contains the minimum.

^c^Children who reported they had experienced any forms of cyberbullying victimization at least once during this semester were classified as the victims of cyberbullying.

CLBC, children who are currently left-behind; PLBC, children who were previously left-behind; NLBC, children who are never left-behind.

### 4.3 Path analysis

**Figure 2:**
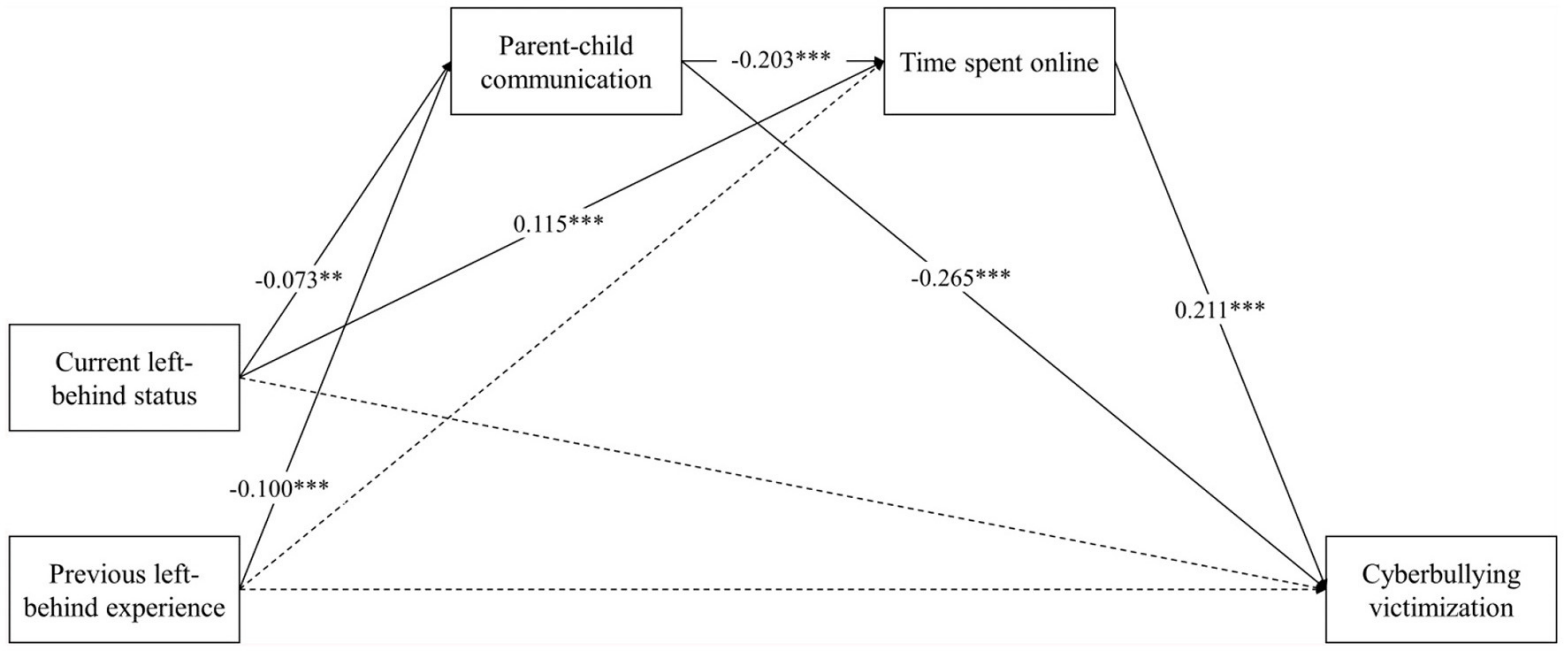
Standardized solutions for the structural model of parental migration, parent-child communication, time spent online, and cyberbullying victimization. Solid lines represent there was statistical significance and dashed lines represent there was no statistical significance. Sex and grade have been controlled. **p < 0.01; ***p < 0.001.

Then, the integrative model was examined. The path analysis revealed that the current left-behind status was associated with higher prevalence of cyberbullying victimization among children (β = 0.076, *p* = 0.024), while the previous experience of being left-behind did not demonstrate a statistically significant correlation (β = 0.058, *p* = 0.087). Regarding the examination of mediating effects, the current status of being left-behind was associated with worse parent-child communication, which, in turn, predicted a higher prevalence of cyberbullying victimization among children [β = 0.019, 95% CI = (0.007, 0.036)]. Likewise, the previous experience of left-behind was associated with worse parent-child communication, which, in turn, predicted a higher prevalence of cyberbullying victimization [β = 0.026, 95% CI = (0.013, 0.043)]. Moreover, the current left-behind status was associated with increased time spent online, which, in turn predicted a higher prevalence of cyberbullying victimization among children [β = 0.024, 95% CI = (0.013, 0.038)]. However, no independent mediating effect of time spent online was observed between previous left-behind experience and cyberbullying victimization [β = 0.006, 95% CI = (−0.004, 0.016)]. Additionally, the current left-behind status positively predicted cyberbullying victimization among children through the serial mediating roles of parent-child communication and time spent online [β = 0.003, 95% CI = (0.001, 0.006)]. Similarly, previous left-behind experience positively predicted cyberbullying victimization among children through the serial mediating roles of parent-child communication and time spent online [β = 0.004, 95% CI = (0.002, 0.007)]. The standardized coefficients are presented in Figure 2 and Table 4.

**Table 4:** Total, direct, and indirect effects of parent-child communication and time spent online in the relationship between current left-behind status or previous left-behind experience and cyberbullying victimization.

	**β**	***p*-value**	**95% CI**	
			**Lower 2.5%**	**Upper 2.5%**
**Total effect**				
Current left-behind status → Cyberbullying victimization	0.076	0.024	0.009	0.143
Previous left-behind experience → Cyberbullying victimization	0.058	0.087	−0.007	0.127
**Direct effect**				
Current left-behind status → Cyberbullying victimization	0.030	0.362	−0.035	0.091
Previous left-behind experience → Cyberbullying victimization	0.022	0.503	−0.040	0.089
**Indirect effect**				
Current left-behind status → Parent-child communication → Cyberbullying victimization	0.019	0.009	0.007	0.036
Current left-behind status → Time spent online → Cyberbullying victimization	0.024	< 0.001	0.013	0.038
Current left-behind status → Parent-child communication → Time spent online → Cyberbullying victimization	0.003	0.012	0.001	0.006
Previous left-behind experience → Parent-child communication → Cyberbullying victimization	0.026	< 0.001	0.013	0.043
Previous left-behind experience → Time spent online → Cyberbullying victimization	0.006	0.259	−0.004	0.016
Previous left-behind experience → Parent-child communication → Time spent online → Cyberbullying victimization	0.004	0.001	0.002	0.007

Sex and grade have been controlled.

## 5 Discussion

### 5.1 Hypothesis model

The current study explored the influence of parental migration on child cyberbullying victimization, and potential mechanisms. Four major findings were generated from our analyses: first, the current status of being left-behind increases the risk of experiencing cyberbullying among children. Second, parent-child communication can mediate the association between current status or previous experiences of being left-behind and cyberbullying victimization among children. Third, the current left-behind status is linked to time spent online, which, in turn, positively correlates with cyberbullying victimization among children. Fourth, parent-child communication and time spent online play serial mediating roles in the association between the current status or previous experiences of being left behind and cyberbullying victimization among children.

The current left-behind status is associated with a higher prevalence of cyberbullying victimization, making CLBC a group that requires particular attention in preventing cyberbullying victimization. In contrast to PLBC, who have already reunited with their migrant parents, CLBC confront the absence of their parents. Face-to-face parent-child interaction is dramatically decreased with the long parent-child separation, and the parent-child communication of LBC is inevitably affected (4, 55). As noted, parents are important channels for children to acquire appropriate Internet knowledge or skills. Good parent-child communication can protect children from cyberbullying victimization (10, 56) by guiding their media use to reduce their susceptibility to engaging in risky activities (57). Impaired parents-child communication can also put LBC in a disadvantaged situation. Migrant parents may have to adopt a lighter touch on LBC's Internet usage because of the time-space span resulting from parent-child separation (58). Without proper parental supervision and monitoring, CLBC were more likely to become addicted Internet users than their counterparts because of longer time spent online (53), and they are more likely to experience cyberbullying (59, 60). Furthermore, for CLBC, functioning as a communication bridge between migrant parents and them, the Internet has already integrated into their daily life, and their time spent online is naturally longer than NLBC, which further urges proper parental supervision and monitoring.

For children who were previously left-behind and are now living with their parents again, parents can actively manage their children's internet usage. Nonetheless, parent-child communication still acts as a mediator between previous left-behind experience and increased risk of cyberbullying victimization among children. This may be attributed to the enduring impact of the parent-child separation on parent-child communication even after children reunite with their parents, as immediate improvement may not occur. This challenge may arise from parents facing their children's altered feelings and emotional needs compared to before the separation (61). Previous research similarly suggests that the previous left-behind experience affects the parent-child communication after reunification, thereby increasing mental health risks among children (62). Moreover, poor parent-child communication increases the likelihood that LBC will rely on the internet for psychological fulfillment. As a result, spending more time online will further increase their susceptibility to cyberbullying.

### 5.2 Theoretical and practical implication

Our study extends the application of ecological systems theory to investigate cyberbullying victimization among children within the context of parental migration. As a crucial environment factor in children's development, the current separation of children from their migrant parents can influence parent-child communication, thereby increasing the risk of children becoming victims of cyberbullying. Importantly, the disruptive nature of intra-family communication persists even after migrant parents are reunited with their children. The lasting impairment of parent-child communication leads children to desire more online interaction, consequently elevating their risk of victimization.

Based on our findings, we propose several implications for intervention targeting cyberbullying victimization among LBC. Parent-child communication appears to be an essential for protecting both CLBC and PLBC from cyberbullying victimization. For children currently left-behind, migrant parents can use mediated and other long-distance communication devices to engage in communication with their children. These long-distance communication can help bridge the spatial gap between family members (63, 64), as indicated by the compensation theory (65). Research has demonstrated that migrant parents can establish positive communication with their children through tools such as video calls (66). Smart-phones offer new spaces for LBC, which can serve as an important supplement to emotion socialization of them (67). Parents often prioritize LBC's academic performance over concerning their emotional needs during telephone conversations (68). Therefore, when leveraging information and communication technologies to enhance frequency of interaction, it is prime for migrant parents to make an effort to address their children's psychological needs. Additionally, for CLBC, due to the absence of parents, managing their digital device usage is crucial. Reducing their internet usage time can help lower the risk of cyberbullying victimization. It might be a good option for migrating parents to turn their children's mobile phones into the Youth Mode (a settable mode that can effectively help parents manage the time of children's Internet access) regardless of the time-space span resulting from parent-child separation. For CLBC whose both parents are absent, caregivers should acquire knowledge of Internet usage to assist children in accessing the internet appropriately. In rural China, there is a scarcity of entertainment facilities, and children have relatively few playmates. As a result, children without their parents present rely on smartphones for entertainment (67). It is essential for the government to implement measures to enhance recreational activities in rural areas, offering more opportunities for children to engage in activities beyond the digital entertainment. For children who were once left behind, it is more important to take effective measures to strengthen parent-child communication upon their parents' return, reshape the parent-child relationship, in order to reduce the risk of children being victims of cyberbullying. To establish intimate parent-child communication, migrated parents can take relevant courses such as parent effectiveness training to polish their parent-child communication skills (69).

Recently, the Chinese government revised the *Law on Protection of Minors* and introduced a new chapter on online protection to safeguard the rights of minors in cyberspace. It proposed measures to enhance the promotion and education of minors' digital literacy across the national, social, school, and family levels. At the family level, it is proposed that parents or other guardians of minors should regulate their own behavior of accessing the Internet and reasonably arrange the time spent online of minors. The current study emphasizes that parents can mitigate the risk of cyberbullying victimization among children by strengthening parent-child communication. Particularly, tailored measures need to be taken to promote the development of children in response to the differing circumstances of parental absence, both current and previous.

### 5.3 Limitations and future research directions

Several limitations should be mentioned. First, the cross-sectional design can only examine associations rather than causal relationships. Subsequent studies applying longitudinal design to validate these associations would be especially meaningful. Second, we did not consider whether the LBC were left behind by both parents or just one, as well as their living with grandparents. Future research should take these factors into account to gain a more comprehensive understanding and clarify the relationship between parental migration and child cyberbullying victimization. Third, the key variables in the current study were based on students' self-reports, which may introduce reporting bias. However, one previous study noted that no difference in effect size was addressed between studies using only self-reports and those using other reports and self-reports simultaneously (14). Fourth, the study sample was limited to children in two provinces of China, which may affect the generalizability of our findings, especially the prevalence of cyberbullying victimization among children. Fifth, the children's time spent online measured in this study did not distinguish academic usage from entertainment usage.

## 6 Conclusions

The present study demonstrated that current left-behind status is associated with an increased risk of cyberbullying victimization among children and that parent-child communication and time spent online independently and sequentially mediate this relationship. Moreover, previous left-behind experience is indirectly related to an elevated risk of cyberbullying victimization among children through poor parent-child communication, which also contributes to extended time spent online, further increased children's vulnerability to cyberbullying. The results stress the vital role of parents in reducing or avoiding children victimization. Interventions aiming at improving the communication frequency and quality between parents and children can exert great benefits on children whose parents currently migrating or previously migrated. It is also necessary to manage their Internet use as the popularity of Internet use in children's lives.

## Data availability statement

The raw data supporting the conclusions of this article will be made available by the authors, without undue reservation.

## Ethics statement

The studies involving humans were approved by the Ethics Committee of the School of Public Health at Zhejiang University. The studies were conducted in accordance with the local legislation and institutional requirements. Written informed consent for participation in this study was provided by the participants' legal guardians/next of kin.

## Author contributions

HZ conceptualized the study conception and design. MW, JL, and XX collected the data. MW and HZ conceived data interpretation and performed all statistical analyses. GZ were responsible for the survey supervision. MW drafted manuscript while HZ, JL, XX, and GZ critically revised the manuscript for important substantial revisions. All authors read and approved the final manuscript.
